# Canada Lancet

**Published:** 1863-05

**Authors:** 


Canada Lancet.—We are most happy to place upon our exchange list the Canada *Lancet,* published in Montreal, and edited by William Edward Bowman, M. D. The British American *Journal* has just suspended for lack of support. This could not, and did not, grow out of any defect in the journal itself, but was rather on account of the high appreciation of the value of a dollar entertained by the physicians of Canada. We hope this new enterprise by Dr. Bowman may be more fortunate, and though the *Lancet* is as yet young and small, it is vigorous and smart, and we think cannot fail for want of support.

